# Variability of obesity prevalence in US states: are obesity growth or deterioration rates constant for different income quantiles?

**DOI:** 10.3389/fepid.2026.1821610

**Published:** 2026-07-08

**Authors:** Yuval Arbel, Yifat Arbel, Oriya Kerner, Miryam Kerner

**Affiliations:** 1 Sir Harry Solomon School of Economics and Management, Western Galilee College, Acre, Israel; 2 Department of Mathematics, Bar Ilan University, Ramat Gan, Israel; 3 The Ruth and Bruce Rapoport Faculty of Medicine, Technion – Israel Institute of Technology, Haifa, Israel; 4 Kaplan Medical Center, Rehovot, Israel

**Keywords:** externalities, health disparities, income quantiles, inequality, obesity

## Abstract

**Background:**

Obesity remains a major public health concern in the United States, with evidence suggesting that its prevalence varies significantly across socioeconomic groups. This study investigates how annual changes in obesity rates differ by income quantiles across U.S. states from 2012 to 2020.

**Methods:**

An empirical model was developed incorporating income-quantile dummy variables and a linear time trend, allowing for differences in average annual changes in obesity prevalence across income groups. Annual change is defined as the year-to-year percentage change in age-adjusted obesity prevalence relative to the preceding year. The analysis draws on state-level data to evaluate baseline differences and subsequent yearly changes in obesity prevalence.

**Results:**

Obesity prevalence differs substantially across income groups in the baseline year, with lower-income groups exhibiting higher prevalence levels. All income groups experienced positive annual increases in obesity prevalence over the study period. Differences in annual changes among lower- and middle-income categories are relatively small in magnitude, while the highest income group (annual income above $75,000) exhibits a larger average annual increase relative to other groups. The estimated time trend is not statistically significant, indicating stable differences in annual changes across income groups over time.

**Conclusion:**

The findings highlight persistent socioeconomic differences in obesity prevalence across U.S. states. While lower-income groups bear a higher overall obesity burden, rising obesity prevalence is observed across the entire income distribution. These patterns suggest that public health responses may benefit from accounting for income-related differences in obesity prevalence, particularly through voluntary approaches such as information campaigns, expanded access to healthy foods and opportunities for physical activity, and initiatives related to the built environment.

## 1 Introduction

The obesity pandemic is one of the most prominent epidemics of the 21st century and is a significant concern among public policy planners. The United States stands out as one of the leading countries globally in terms of obesity rates. The rapid growth of obesity in the U.S. is particularly alarming. Back in 1990, there was not a single U.S. state with an obesity rate exceeding 15%, whereas by 2015 every state had rates surpassing 20% (1, 2). More recent surveillance data indicate that obesity prevalence has continued to rise, with substantial variation across states and population subgroups (3, 4).

Income level is a key factor associated with differences in obesity prevalence, as numerous studies show. Research has examined this relationship at both national and within-country levels, comparing income disparities between rich and poor populations. In addition to income, obesity prevalence in the United States has been shown to vary systematically by education, race and ethnicity, and place of residence. Adults with lower educational attainment, non-Hispanic Black and Hispanic populations, and residents of rural areas consistently exhibit higher obesity prevalence than their respective counterparts (3, 4). These disparities highlight the multifactorial nature of obesity, associated with differences in food environments, healthcare access, occupational and leisure-time physical activity, psychosocial stress, and local policy contexts.

Analyzing data from 147 countries over a 40-year period (1975–2014), Talukdar et al. (5) found that a 1% increase in a country's national income is associated with a 1.32% rise in obesity prevalence among men and a 1.01% increase among women. This indicates that as a country's income level rises, so does its obesity prevalence at the macroeconomic level.

However, this relationship is reversed when obesity prevalence is examined across income groups within Western countries. Bentley et al. (1) document an inverse relationship between obesity prevalence and income levels in the United States over the past three decades. In 2015, the Pearson correlation between the natural logarithm of obesity prevalence and income was −0.699, compared with a substantially weaker correlation in 1990, suggesting that the income–obesity gradient has strengthened over time.

While the inverse association between income and obesity prevalence in the United States is well documented (1, 3, 4), most studies focus on obesity prevalence levels and socioeconomic disparities at a given point in time. Research has also documented long-term trends in obesity prevalence across populations and geographic areas (2, 5). However, comparatively little evidence is available regarding whether annual changes in obesity prevalence differ systematically across income groups within the United States. Consequently, it remains unclear whether income-related disparities are stable over time or whether some income groups are experiencing more rapid increases in obesity prevalence than others. From a policy perspective, understanding these dynamics is important because groups with similar current prevalence levels may face substantially different future obesity burdens if their rates of change differ.

To address this gap, the present study examines annual changes in obesity prevalence across income quantiles and U.S. states. Using data from the Centers for Disease Control and Prevention (CDC) (6), the empirical model incorporates annual percentage changes in obesity prevalence as the dependent variable and includes income-quantile indicators and a linear time trend. This structure allows for the identification of differences in average annual changes across income groups while assessing whether these differences vary over time.

The analysis relies on state-level obesity prevalence derived from the Behavioral Risk Factor Surveillance System (BRFSS), which is based on self-reported height and weight. While self-reported data are known to underestimate true obesity prevalence relative to objectively measured data (7, 8), prior validation studies indicate that this reporting bias is relatively stable over time (9–11). Because the present study focuses on annual changes in obesity prevalence rather than prevalence levels, time-invariant components of reporting bias are differenced out, rendering the estimated trends less sensitive to systematic misreporting. The BRFSS employs a complex survey design with sampling weights and age-standardization procedures to produce state-level prevalence estimates, and our analysis relies on these published, population-weighted measures when constructing annual changes by income category. A formal treatment of measurement error under the first-difference specification is provided in Appendix A.

This study contributes to the literature on socioeconomic disparities in obesity by shifting attention from obesity prevalence levels to obesity growth dynamics. While prior research has extensively documented income-related differences in obesity prevalence and long-run obesity trends, less is known about whether obesity prevalence is increasing at similar rates across income groups within the United States. Using state-level data from the CDC for the period 2011–2020, this study examines income-specific differences in annual changes in obesity prevalence. By focusing on obesity growth rates rather than prevalence levels, the analysis provides new evidence on whether income-related disparities are widening, narrowing, or remaining stable over time. The study is descriptive in nature and does not seek to establish causal relationships; rather, it documents income-specific patterns in obesity dynamics that may inform future research and policy discussions.

The remainder of the paper is organized as follows. Section 2 presents the data and descriptive statistics, Section 3 outlines the empirical methodology, Section 4 reports the results, Section 5 discusses the findings and their policy implications, and Section 6 concludes.

## 2 Descriptive statistics

This study uses state-level obesity data obtained from the Centers for Disease Control and Prevention (CDC) (6). The dataset covers the period 2011–2020. The analysis ends in 2020 because this was the most recent year available in the harmonized dataset used in this study, ensuring consistent measurement of obesity prevalence across states, income categories, and years.

A key feature of the dataset is the availability of state-level obesity prevalence estimates stratified by household income. Income categories follow the standard BRFSS definitions: <$15,000, $15,000–$24,999, $25,000–$34,999, $35,000–$49,999, $50,000–$74,999, and ≥$75,000. These categories are defined consistently throughout the study period. Details regarding panel construction and data availability by income group are provided in Appendix Table B1.

The unit of analysis is a state–income–year cell. For each state, the CDC reports age-adjusted obesity prevalence separately for each household income category and year. Thus, a single observation corresponds to the obesity prevalence of a particular income group in a specific state and year. The dependent variable is subsequently constructed as the year-to-year percentage change in obesity prevalence within each state–income category. Because annual changes require a lagged value, observations from 2011 are used only to calculate changes for 2012 and are not included in the estimation sample. The resulting panel therefore consists of repeated observations on state–income cells over time.

The dependent variable is the annual percentage change in obesity prevalence. Consequently, observations from 2011 are used only to construct year-to-year changes and are not included in the estimation sample. Additional observations are unavailable for a limited number of state–income–year combinations due to missing obesity prevalence estimates. Appendix Table B2 summarizes the resulting estimation sample (*N* = 2,355) and documents all observation losses.

The estimation sample consists of 53 jurisdictions observed across five income categories and nine annual changes (2012–2020). A fully balanced jurisdiction contributes 45 observations (5 income categories   ×   9 annual changes), yielding 2,355 observations after accounting for a small number of missing income-specific prevalence estimates in New Jersey, Puerto Rico, and Guam.

Obesity prevalence is measured using age-adjusted, income-specific state-level estimates reported by the CDC. Age adjustment is performed through direct standardization to a fixed reference population, thereby enhancing comparability across states, income groups, and years. Because the analysis focuses on within-state changes over time, this procedure minimizes differences attributable to variation in age composition while preserving the variation relevant to the analysis. All descriptive statistics and regression estimates are based on these aggregated prevalence measures, with statistical inference adjusted for within-state correlation over time.

Table 1 presents descriptive statistics for the main variables. Average obesity prevalence across all states and years is 31.58%, with a standard deviation of 5.26 percentage points. Values range from 12.1% to 50.5%, indicating substantial variation across states. The annual percentage change in obesity prevalence (*Δ*) averages 2.56%, although considerable variability is observed, with values ranging from −38.4% to 91.7%.

**Table 1:** Descriptive statistics of selected variables.

Variable	Description	Obs	Mean	Std.	Min	Max
Obesity prevalence	Percent of obese population at a statewide level	3,150	31.58	5.26	12.1	50.5
Delta	the annual percent of change in obesity prevalence calculated as $\frac{Obesity\_per-LAG(Obesity\_Per)}{LAG(Obesity\_Per)}$	2,826	0.0256	0.1145	−0.3838	0.9172

The conventional measure of obesity is $BMI=\frac{Kg}{meter^{2}}\geq 30$ where Kg and meter reflects the persons’ weight and height, respectively.

The annual change in obesity prevalence (*Δ*), defined as the year-to-year percentage change relative to the preceding year, has a mean of 2.56 percent and a standard deviation of 11.45 percent. The distribution of *Δ* exhibits considerable dispersion, with observed values ranging from a decline of approximately 38 percent to an increase of approximately 92 percent.

The relatively wide range of annual changes reflects both the sensitivity of percentage-growth measures to baseline prevalence levels and the fact that the underlying obesity estimates are derived from survey data. Consequently, relatively large percentage changes may occur when changes are measured relative to comparatively low baseline prevalence values. For this reason, mean values are supplemented with additional distributional statistics in Table 2, including medians, interquartile ranges, skewness, and kurtosis, which provide a more complete characterization of the distribution of annual obesity changes.

**Table 2:** Extended descriptive statistics of annual changes in obesity prevalence (*Δ*) by income group.

Income category	Obs.	Mean	Median	IQR	Std.	Skewness	Kurtosis
< $15,000	471	0.0229	0.0106	0.161	0.1369	0.7949	5.388
$15,000–$24,999	471	0.0207	0.0204	0.122	0.0971	0.4149	3.7747
$25,000–$34,999	471	0.027	0.0192	0.1469	0.1264	0.7702	5.5345
$35,000–$49,999	471	0.0271	0.0213	0.1413	0.1131	0.4394	4.7025
$50,000–$74,999	471	0.0256	0.0219	0.132	0.1103	0.1145	3.9834
$75,000 or greater	471	0.03	0.0286	0.1039	0.0983	1.7789	19.6813
Total/Mean	2,826	0.0256	0.0205	0.1327	0.1145	0.7113	6.6184

*Δ* denotes the annual change in obesity prevalence. Median corresponds to the 50th percentile. The interquartile range (IQR) is calculated as the difference between the 75th and 25th percentiles. Skewness and kurtosis are based on Stata `summarize, detail` output. Each income group contains 471 observations, reflecting a balanced panel across states and years with non-missing *Δ*.

Although the highest-income category exhibits substantial kurtosis, the robustness analysis reported in Section 4.2 and Appendix Table D1 demonstrates that the main regression results remain qualitatively unchanged when *Δ* is winsorized at the 1st and 99th percentiles. This suggests that the observed income-gradient patterns are not driven by a small number of extreme observations.

Table 2 reports extended descriptive statistics for the annual change in obesity prevalence (*Δ*) by income category. Mean annual changes are positive across all income groups, ranging from approximately 2.1 to 3.0 percent. Median changes are smaller than mean values in all income categories, indicating right-skewed distributions of annual changes. Dispersion varies across income groups, as reflected in differences in standard deviations and interquartile ranges. The highest income category ($75,000 or greater) exhibits both the largest mean annual change and substantially higher skewness and kurtosis, suggesting occasionally extreme positive changes in obesity prevalence.

The highest-income category ($75,000 or greater) exhibits substantial positive kurtosis (19.68), indicating a distribution with heavy tails and occasional extreme changes in obesity prevalence. While this pattern suggests the presence of outlying observations, the similarity between the mean and median indicates that the overall trend is not dominated by a small number of extreme values. Because these statistics are descriptive, the observed kurtosis does not in itself compromise the validity of the subsequent panel-data analysis.

**Figure 1:**
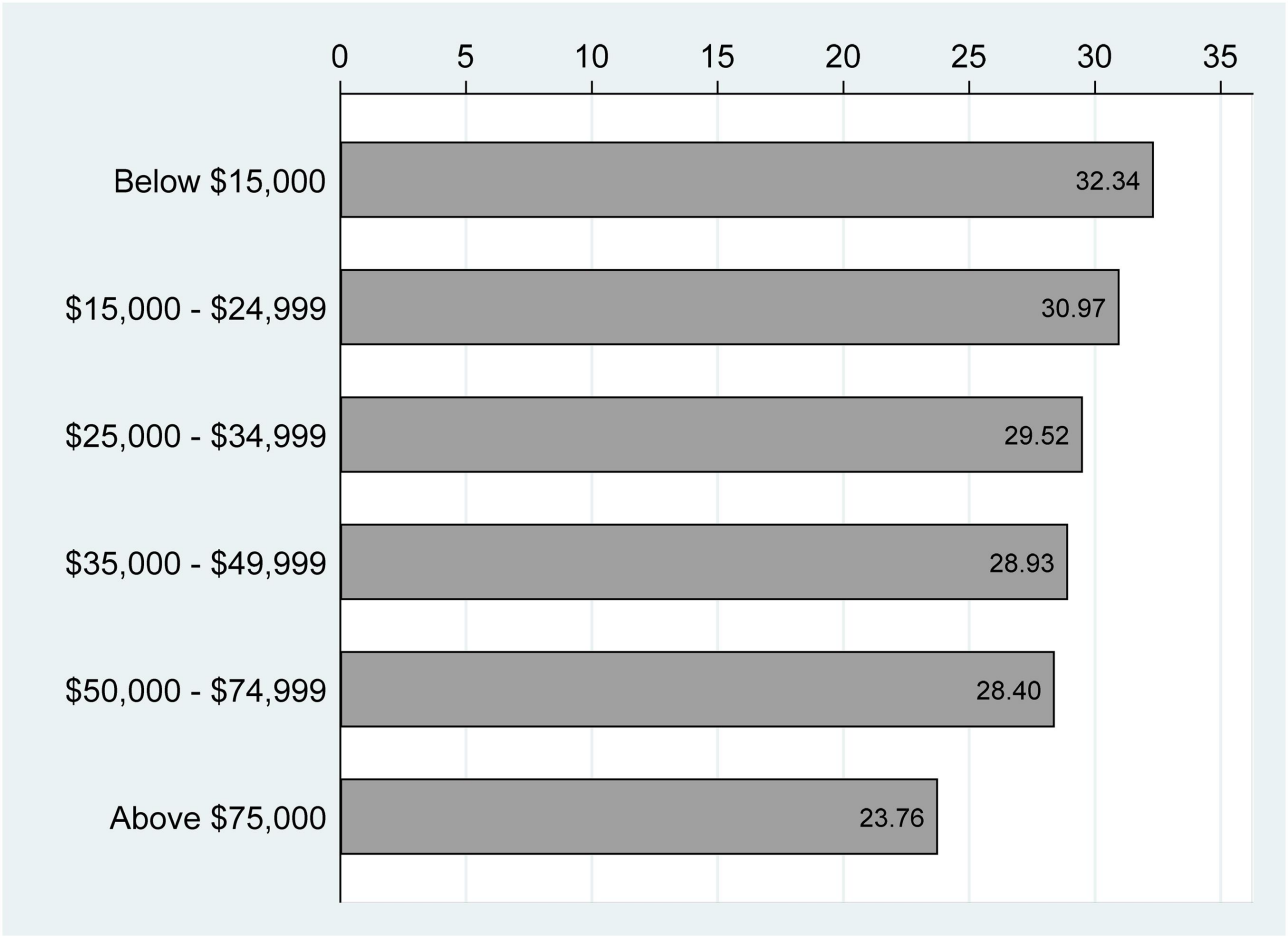
Obesity prevalence (%) by household income category (2011).

Figure 1 presents mean obesity prevalence in the baseline year (2011), stratified by income category. A clear negative association between income and obesity prevalence is observed. In the lowest income category (annual household income below $15,000), 32.34 percent of the population is classified as obese, compared with 23.76 percent in the highest income category (annual household income above $75,000).

**Figure 2:**
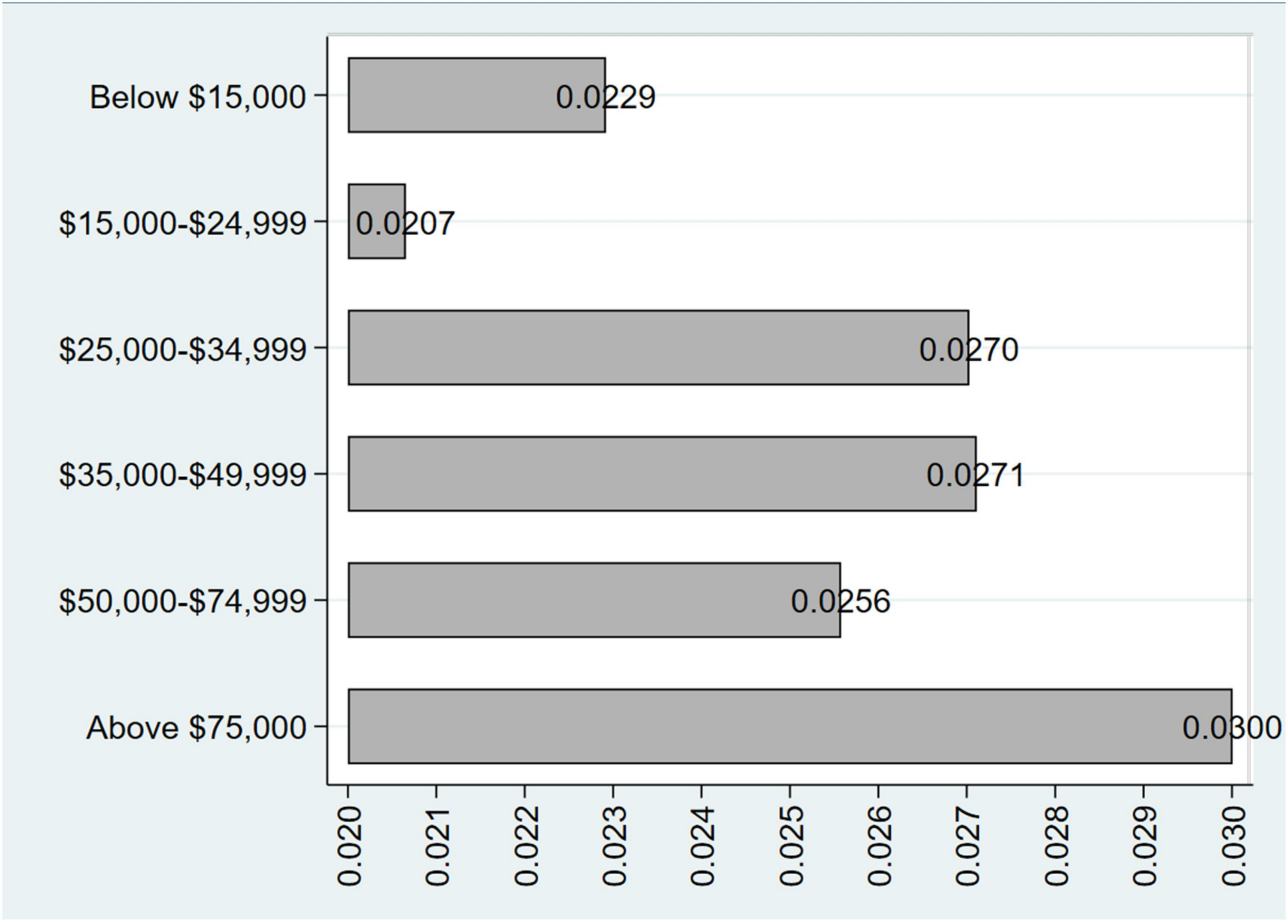
Average annual modification in obesity prevalence 2012-2020. One year is lost in Figure 2 due to the construction of annual changes.

Figure 2 depicts the average annual change in obesity prevalence over the period 2012–2020, by income category. Average annual changes are positive across all income groups, with magnitudes that vary by income level. Lower- and middle-income categories exhibit similar average annual changes, while the highest income category displays a larger average annual change. As noted above, one year is lost in the construction of annual changes due to the lagged definition of *Δ*.

## 3 Methodology

### 3.1 Outcome variable

Let $\Delta_{i,t}$ denote the annual percentage change in obesity prevalence in state *i* between years t−1 and *t*, defined as:

$$\Delta_{i,t}=\frac{\mathrm{Obesit}y_{i,t}-\mathrm{Obesit}y_{i,t-1}}{\mathrm{Obesit}y_{i,t-1}}.$$

Obesity prevalence is measured as the percentage of adults classified as obese at the state level, based on age-adjusted estimates reported by the Centers for Disease Control and Prevention (CDC) (6). Because annual percentage changes require a lagged prevalence measure, observations from 2011 are used solely to construct year-to-year changes and are excluded from the estimation sample. Consequently, the estimation period for $\Delta_{i,t}$ spans 2012–2020.

### 3.2 Model specification

To examine differences in obesity growth rates across household income groups, we estimate the following linear regression model:

$$\Delta_{i,t}=\alpha +\overset{5}{\underset{k=2}{\sum }}\;\beta_{k}D_{i,k}+\gamma t+\varepsilon_{i,t},$$

where $D_{i,k}$ is a dummy variable equal to one if state *i* belongs to income category *k*, and zero otherwise, with k=2,…,5. The baseline category (k=1) corresponds to households with annual incomes of $15,000–$24,999. The remaining categories represent incomes of $25,000–$34,999, $35,000–$49,999, $50,000–$74,999, and $75,000 or more. Households with incomes below $15,000 are excluded from the main specification and are examined separately in alternative specifications reported in the Appendix. The variable *t* denotes a linear time trend defined as t=(Year−2012), so that t=0 corresponds to 2012, the first year for which the annual change $\Delta_{i,t}$ is defined. The error term $\varepsilon_{i,t}$ captures unobserved shocks to obesity growth at the state–year level.

### 3.3 Interpretation of coefficients

The coefficients $\beta_{k}$ capture differences in the observed average annual change in obesity prevalence across income groups, relative to the baseline income category ($15,000–$24,999). The intercept α represents the model-implied annual percentage change in obesity prevalence for the reference income group in 2012. The coefficient γ captures a common linear time trend in obesity growth that applies uniformly across income groups.

This specification allows for persistent income-related differences in obesity growth rates while maintaining a parsimonious structure that facilitates transparent interpretation.

### 3.4 Rationale for excluding income-specific time trends

A more general specification could allow for income-specific time trends through interactions between income indicators and the time variable. However, exploratory analysis indicates that such interaction terms are neither individually nor jointly statistically significant. Moreover, because the dependent variable is defined as a year-to-year growth rate, smooth long-run trends in obesity prevalence are already differenced out to a large extent. Allowing for heterogeneous time trends across income groups does not materially improve model fit or alter the estimated income effects.

Accordingly, we adopt the more parsimonious specification above, which captures stable differences in obesity growth across income groups without imposing unnecessary additional structure.

### 3.5 Estimation and inference

The model is estimated using ordinary least squares. Standard errors are clustered at the state level to account for serial correlation and heteroskedasticity within states over time. All reported confidence intervals and hypothesis tests are therefore robust to arbitrary within-state error correlation.

### 3.6 Presentation of results

To facilitate interpretation, we report both regression coefficients and adjusted predictions of annual obesity growth rates by income category. Predicted values are computed from the estimated model and presented graphically with 95% confidence intervals based on clustered standard errors.

## 4 Results

Table 3 reports the regression results based on the empirical model described in Section 3. Column (3) presents estimates from the full specification including income-category indicators and a linear time trend, with standard errors clustered at the state level. Column (4) reports the corresponding stepwise specification excluding the time trend.

**Table 3:** Income differences in the annual growth of obesity prevalence (*Δ*). Dependent variable: Annual percentage change in obesity prevalence (*Δ*). Sample: Income categories $25,000–$34,999 through $75,000 or greater. Reference category: $15,000–$24,999.

Variables	(1) Full model (Robust SE)	(2) Stepwise model (Robust SE)	(3) Full model (Clustered SE)	(4) Stepwise model (Clustered SE)
Constant	0.01851*** (0.00558)	0.02608*** (0.00226)	0.01851*** (0.00325)	0.02066*** (0.00168)
$25,000–$34,999	0.00637 (0.00735)	—	0.00637** (0.00272)	0.00637** (0.00272)
$35,000–$49,999	0.00646 (0.00687)	—	0.00646** (0.00288)	0.00646** (0.00288)
$50,000–$74,999	0.00492 (0.00677)	—	0.00492** (0.00220)	0.00492** (0.00220)
$75,000 or greater	0.00935 (0.00637)	—	0.00935*** (0.00250)	0.00935*** (0.00250)
Time trend (year−2012)	0.00053 (0.00086)	—	0.00053 (0.00061)	—
Observations	2,355	2,355	2,355	2,355
States (clusters)	—	—	53	53
F-statistic	0.55	0.00	2.86**	3.57**
Prob > F	—	—	0.0237	0.0121

Columns (1) and (3) report full models including a linear time trend. Columns (2) and (4) report reduced models selected via stepwise regression with a removal criterion of *p* < 0.05. Column (1) uses heteroskedasticity-robust standard errors, while columns (3) and (4) cluster standard errors at the state level. The omitted income category is household income between $15,000-$24,999. .

Statistical significance is denoted as;

*** *p* < 0.01.

** *p* < 0.05.

Across specifications, the estimated coefficients indicate systematic differences in the average annual change in obesity prevalence across income groups**.** Relative to the reference category ($15,000–$24,999), lower- and middle-income groups exhibit annual changes that are similar in magnitude and not statistically distinguishable from one another. By contrast, the highest income category (annual household income of $75,000 or greater) displays a significantly larger average annual increase in obesity prevalence.

The estimated linear time trend is small in magnitude and not statistically significant**,** indicating that income-related differences in annual obesity changes are stable over time. This finding supports the parsimonious specification without income-specific time trends, as discussed in Section 3.4.

**Figure 3:**
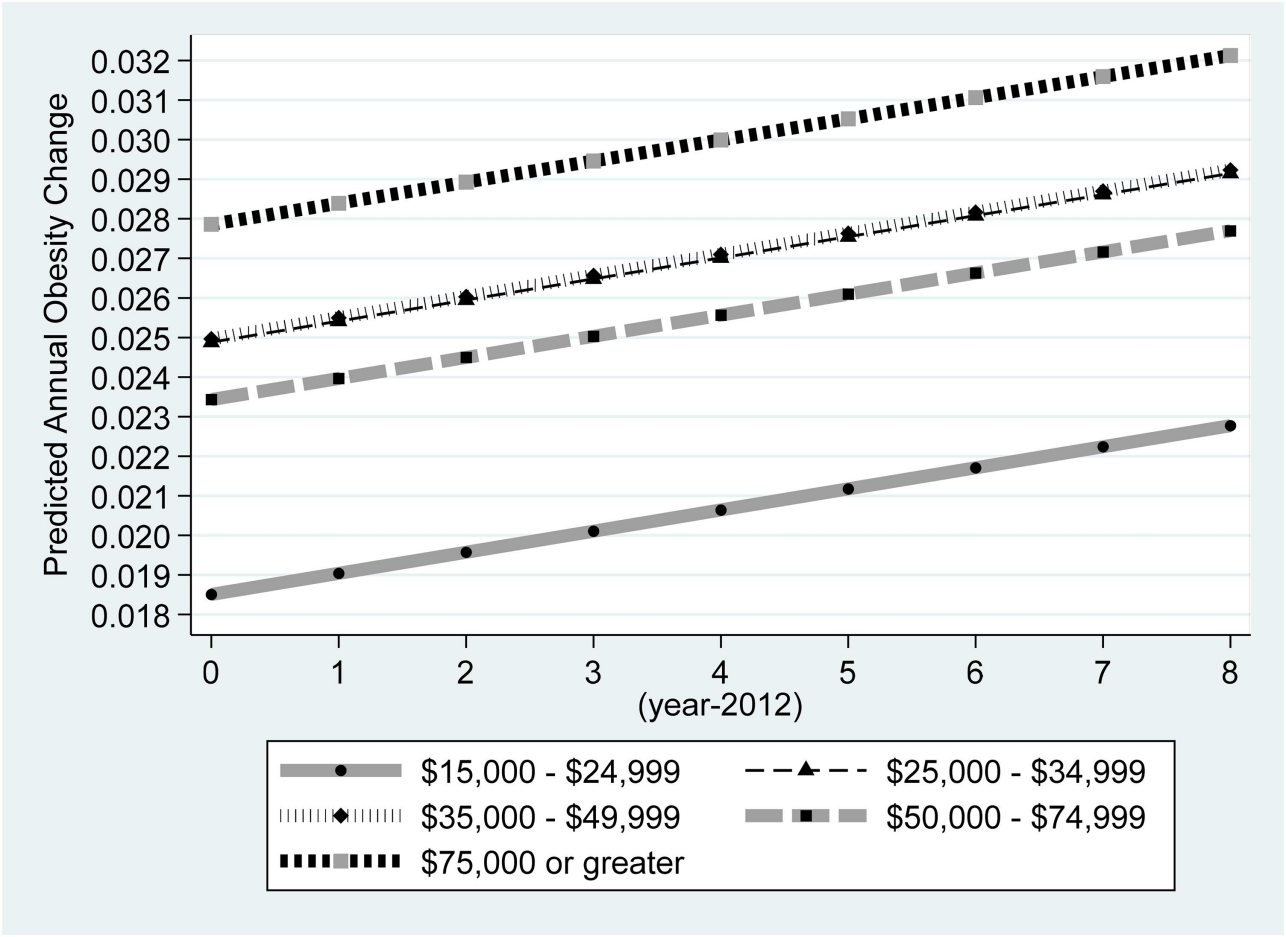
Predicted annual change in obesity prevalence by income category**.** Predicted values are computed from the full specification reported in column (3) of Table 3, which includes income category indicators and a linear time trend (year−2012), with standard errors clustered at the state level. The dependent variable is the annual percentage change in obesity prevalence. The estimated time trend is not statistically significant. Predicted trajectories for the $25,000–$34,999 and $35,000–$49,999 income categories overlap closely over most of the sample period.

Figure 3 presents predicted annual changes in obesity prevalence by income category based on the full specification with clustered standard errors. Predicted trajectories for lower- and middle-income categories overlap closely over the sample period, while the highest income category exhibits consistently larger predicted annual increases.

**Figure 4:**
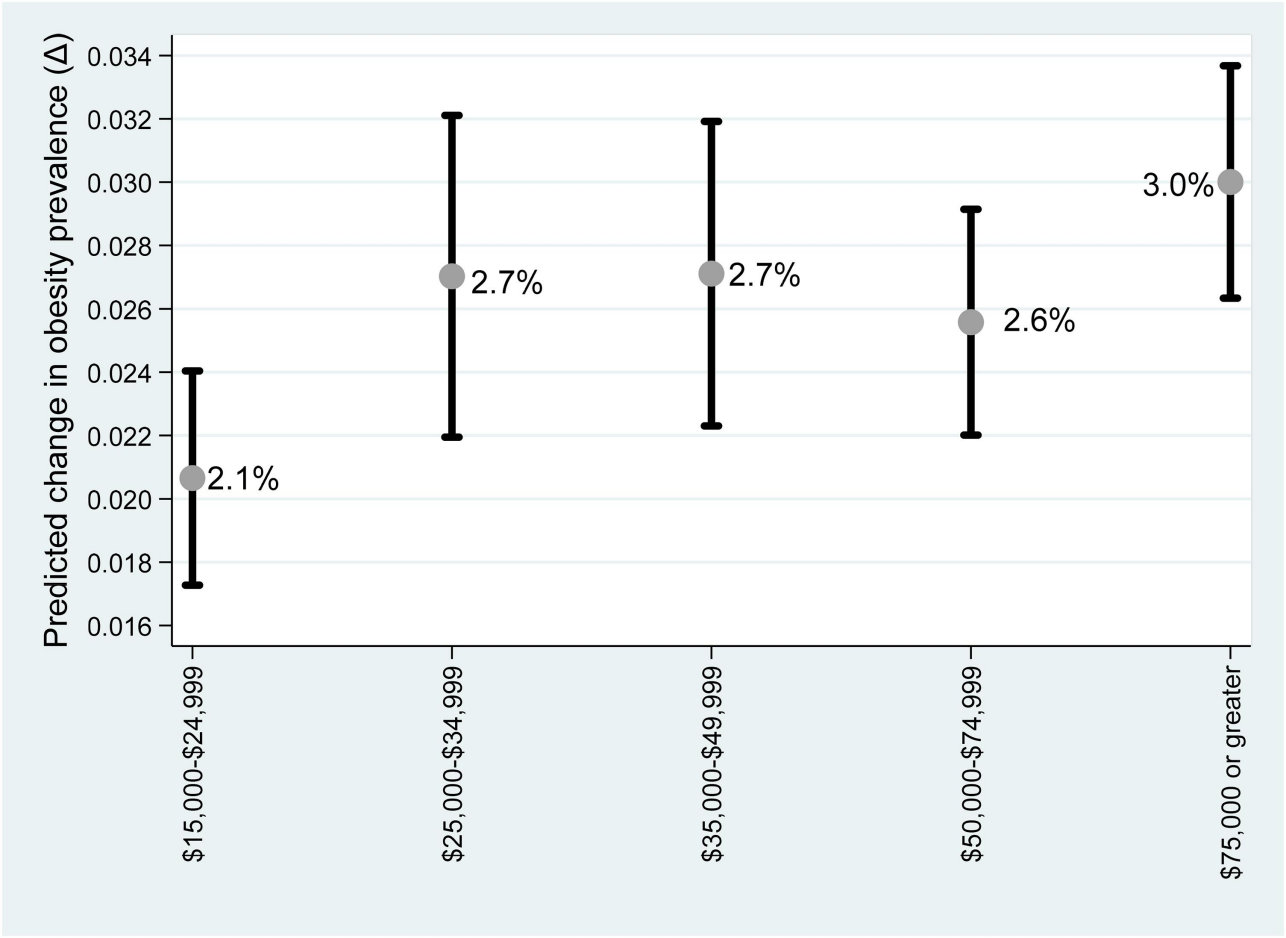
Predicted annual change in obesity prevalence by household income. Predicted annual changes in obesity prevalence by household income category are computed from the stepwise specification reported in column (4) of Table 3, which includes income indicators but excludes the linear time trend. The dependent variable is the year-to-year percentage change in obesity prevalence. Error bars denote 95% confidence intervals based on standard errors clustered at the state level.

Figure 4 reports predicted annual changes based on the stepwise specification. The qualitative pattern remains unchanged: annual obesity increases are positive across all income groups, with the largest increases observed for the highest income category. Differences between the highest income group and other categories are statistically significant at conventional levels.

**Figure 5:**
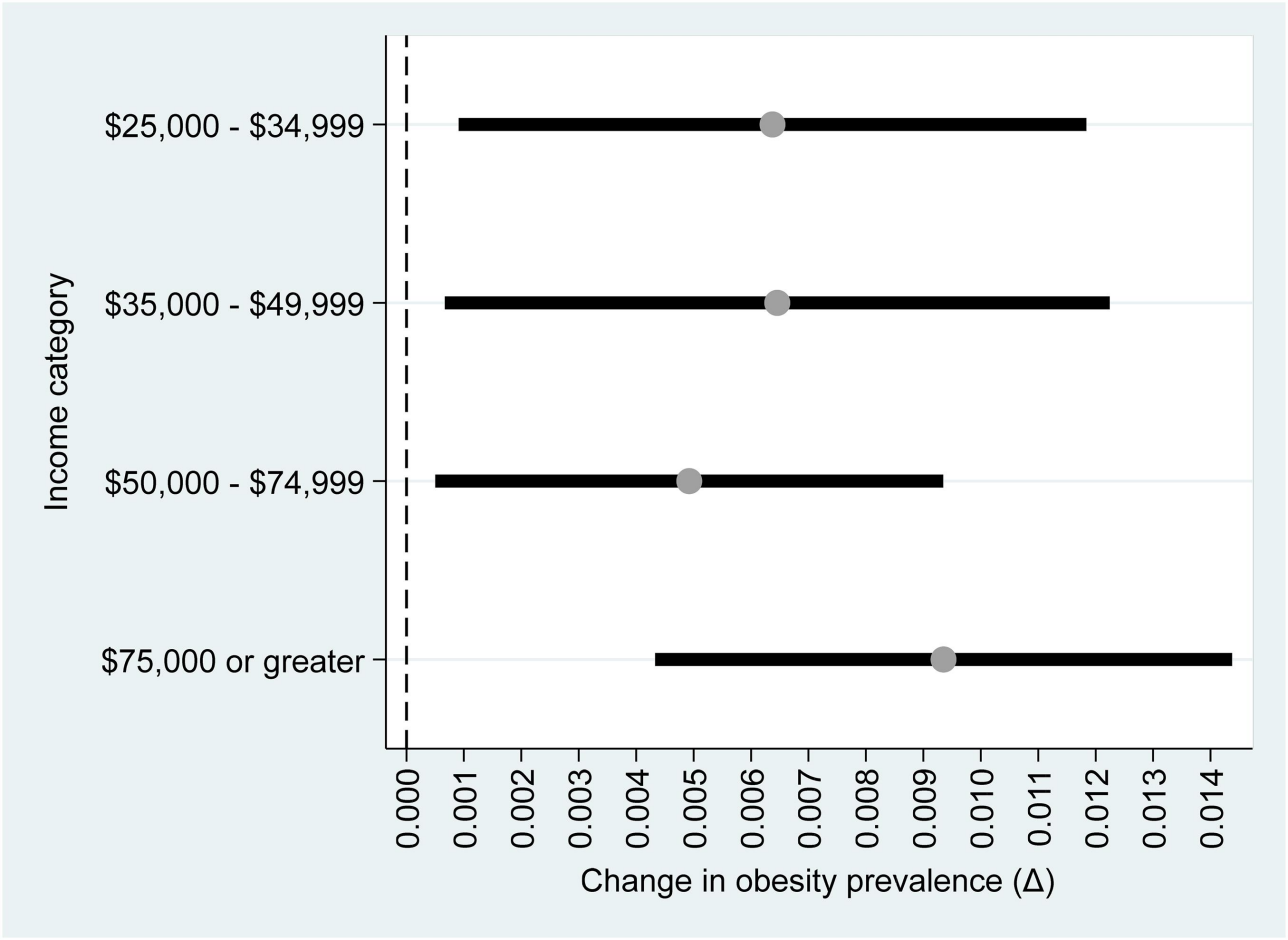
Differences in annual obesity growth relative to the $15,000–$24,999 income group**.** The figure shows estimated differences in the annual growth rate of obesity prevalence across household income categories, relative to the reference group with annual income between $15,000 and $24,999, based on the stepwise specification reported in column (4) of Table 3. Points denote coefficient estimates from a linear regression of the annual percentage change in obesity prevalence, and horizontal bars indicate 95% confidence intervals based on standard errors clustered at the state level. The dashed vertical line at zero denotes no difference relative to the reference category. The estimated annual growth rate of obesity prevalence for the reference group is 2.07%.

Figure 5 summarizes the estimated differences in annual obesity growth relative to the $15,000–$24,999 income group. Consistent with the regression results, only the highest income category exhibits a statistically significant difference in annual obesity growth.

### 4.1 Sensitivity analyses including the lowest income category

Appendix C reports a comprehensive set of robustness and sensitivity analyses that reintroduce households with annual incomes below $15,000 into the estimation sample. The purpose of these analyses is twofold: first, to verify that the main findings are not driven by the exclusion of the lowest income category, and second, to assess whether obesity growth dynamics for this group differ meaningfully from those observed in adjacent income categories.

Appendix Table C1 presents regression estimates for the full income distribution, using households with incomes below $15,000 as the reference category. Across both heteroskedasticity-robust and state-clustered specifications, the estimated coefficients for income groups between $15,000 and $74,999 are small in magnitude and statistically indistinguishable from zero. In contrast, the highest income category ($75,000 or greater) continues to exhibit a significantly larger average annual increase in obesity prevalence. The estimated linear time trend remains statistically insignificant, mirroring the results reported in the restricted sample in the main text.

Appendix Figures C2, C3 further illustrate these patterns using adjusted predictions from the full specification. Predicted annual obesity changes for households earning below $15,000 are nearly identical to those for households earning between $15,000 and $74,999 over the entire sample period. The close overlap of these predicted trajectories indicates that the lowest income category does not follow a distinct obesity growth path relative to other low- and middle-income groups. Consistent with the regression results, only the highest income group displays a visibly higher predicted annual change.

Appendix Figure C4 reports estimated differences in annual obesity growth relative to the below-$15,000 reference group. With the exception of the highest income category, all confidence intervals include zero, confirming that differences in obesity growth between the lowest income group and other non-affluent groups are not statistically meaningful.

Taken together, the results in Appendix C demonstrate that households with incomes below $15,000 exhibit obesity growth dynamics that are empirically indistinguishable from those of adjacent income categories. Their inclusion does not alter coefficient magnitudes, statistical significance, or the qualitative ranking of income groups. As a result, excluding this category from the main specification serves to simplify presentation and interpretation without sacrificing empirical content. Importantly, the key substantive finding—that the highest income group experiences a larger average annual increase in obesity prevalence while lower- and middle-income groups display similar growth rates—remains fully robust to the inclusion of the lowest income category.

### 4.2 Robustness to extreme annual changes

The distribution of annual changes in obesity prevalence exhibits right skewness and occasional extreme values, particularly in higher income groups (Table 2). To assess whether the estimated income gradients are driven by such extremes, we re-estimated the model after winsorizing the annual change measure (*Δ*) at the 1st and 99th percentiles. We conduct this robustness analysis for both the full sample (including all income categories) and the restricted sample excluding the lowest income group, using the same specification as in the baseline regressions. Across both samples, the estimated income effects retain their sign, relative magnitude, and statistical significance. The corresponding robustness results are reported in Appendix Table D1.

### 4.3 Percentage-point changes in obesity prevalence

As a robustness check addressing the bounded nature of obesity prevalence and potential heteroskedasticity in percent changes, we re-estimated the main specification using the annual percentage-point change in obesity prevalence as the dependent variable. Results are reported in Appendix Table E1 for both the restricted estimation sample (income categories $15,000–$24,999 through $75,000 or greater) and the full sample including households with incomes below $15,000.

Across both specifications, the estimated income-group coefficients display the same qualitative pattern as in the percent-change models. Annual obesity increases are smallest for lower- and middle-income groups and largest for the highest income category ($75,000 or greater), while differences among lower income categories are not statistically distinguishable. The estimated linear time trend remains statistically insignificant.

Although the magnitudes of the coefficients differ due to the alternative scale of the dependent variable, the relative ordering and substantive interpretation of income-group differences are unchanged. These results indicate that the main findings are not driven by the choice of percent-change versus percentage-point change in obesity prevalence.

The primary outcome is the annual percentage change in obesity prevalence because it measures relative growth and facilitates comparisons across income groups with different baseline prevalence levels. Percentage-point changes are reported as a robustness analysis and produce substantively similar conclusions.

## 5 Discussion and policy implications

Obesity prevalence remained substantially higher among lower-income households throughout the study period, while annual increases in obesity prevalence were observed across all income groups. The descriptive statistics and regression results indicate that differences in obesity growth rates among low- and middle-income categories were relatively small. In contrast, the highest-income category exhibited a modest but statistically significant increase in annual obesity growth relative to the reference group. However, the magnitude of this difference was small in absolute terms, suggesting that obesity prevalence is increasing broadly throughout the income distribution rather than being concentrated within a particular socioeconomic group. These findings indicate that income-related disparities in obesity persist, but that differences in annual obesity growth rates are considerably less pronounced than differences in obesity prevalence levels.

Another important finding is the absence of a statistically significant time trend in obesity growth rates. This result suggests that the relative differences in annual obesity growth across income groups remained largely stable during the study period. Consequently, while income disparities in obesity prevalence persist, there is limited evidence that these disparities are either widening or narrowing through differential growth rates. While prevalence levels quantify the current burden of obesity, annual growth rates provide complementary information regarding the pace of change and may help identify population groups in which future obesity burdens are likely to increase most rapidly. Taken together, the findings suggest that socioeconomic disparities in obesity are reflected primarily in prevalence levels rather than in sharply divergent growth trajectories. Without implying causal relationships, these descriptive results contribute to a more complete understanding of obesity dynamics across income groups and may help inform the design of income-sensitive prevention and intervention strategies.

The results are most directly informative for voluntary and non-coercive approaches, including information and media campaigns, programs that expand access to healthy foods, initiatives that promote physical activity, and policies related to the built environment. Such strategies operate by shaping knowledge, opportunities, and environments rather than imposing direct behavioral constraints, and they can be tailored to different income groups in light of persistent differences in obesity prevalence. This emphasis is consistent with public health frameworks that highlight environmental and informational mechanisms as central components of obesity prevention (12).

By contrast, targeted fiscal interventions, such as taxes on unhealthy foods, raise distinct efficiency and welfare considerations. Theoretical and empirical studies have shown that the effects of such policies may be ambiguous and depend critically on behavioral responses, substitution patterns, and distributional impacts (13–15).

Consistent with this literature, the present study does not evaluate the effectiveness of specific policy instruments. Instead, it provides empirical evidence on the distribution and evolution of obesity prevalence across income groups that can inform ongoing policy debates and motivate future empirical research on obesity prevention strategies.

## 6 Summary and conclusions

This article analyzes the rate of change in obesity prevalence in the United States between 2011 and 2020, categorized by states and income levels. Relatively few studies have examined income-specific differences in annual changes in obesity prevalence using state-level data. The study relies on CDC data, with a link to the data source provided in the reference list (6). Due to the inclusion of categorical income variables in the data, the proposed model utilizes binary variables defined by income percentiles and time variables. One feature of this approach is that it allows for non-linearity in income categories. As demonstrated by Ameye and Swinnen (16), comparing obesity rates among countries segmented by income levels reveals non-linear trends. In many developing countries, the prevalence of obesity is higher among higher-income individuals, whereas in developed Western countries it is higher among lower-income populations.

The findings show that average annual changes in obesity prevalence are positive across nearly all income brackets, including the highest income category. The analysis does not extend to long-horizon projections beyond the observed sample period. Results are interpreted at the ecological, state level and may reflect compositional confounding and measurement limitations.

Several limitations should be acknowledged. First, the study relies on state-level data for the United States. This level of aggregation does not permit analysis at finer geographic scales, such as urban–rural stratifications or within-state variation in obesity prevalence. It also prevents investigation of individual-level mechanisms linking income and obesity. At the same time, state-level analysis enables systematic comparisons across jurisdictions with broadly similar institutional and cultural contexts. Unlike studies focusing on individual cities without comparative benchmarks (17, 18), this approach permits comparisons across U.S. jurisdictions. Moreover, whereas cross-country analyses (5, 16), face substantial heterogeneity in culture, income levels, occupations, and consumption patterns, comparisons across U.S. states reduce some of these sources of heterogeneity. Accordingly, all descriptive statistics and regression results should be interpreted as state-level ecological associations rather than individual-level relationships.

Second, the analysis treats the published state–income–year obesity prevalence estimates as observed inputs. These estimates are derived from BRFSS survey data and are therefore subject to sampling variability. Because the publicly available CDC data do not consistently provide the information required to propagate survey-estimation variance across all state–income–year cells, uncertainty associated with the original prevalence estimates is not incorporated into the calculation of annual changes or subsequent regression analyses. Consequently, the reported confidence intervals may understate total statistical uncertainty.

Third, the analysis is restricted to 2011–2020, corresponding to the most recent year available in the harmonized dataset used in this study. More recent evidence suggests that obesity prevalence continued to increase after 2020 across several U.S. populations and geographic areas. For example, Shoman et al. (19) documented a continued rise in obesity prevalence among adults in Mississippi between 2017 and 2023, while Agboola et al. (20) reported persistent sociodemographic disparities and high obesity prevalence among U.S. adults during the 2021–2023 period. These findings suggest that the upward trajectory of obesity observed in the present analysis likely persisted beyond the study period. Nevertheless, because the primary objective of this study is to compare relative differences in annual obesity growth rates across income groups rather than to estimate current obesity prevalence levels, the main conclusions are unlikely to be substantially affected. Future research should extend the analysis as additional years of comparable data become available.

Fourth, percentage-change outcomes are sensitive to baseline prevalence levels, such that identical absolute changes may translate into different percentage changes across income groups. However, robustness analyses based on percentage-point changes produced substantively similar conclusions, suggesting that the main findings are not driven by the choice of outcome metric.

Fifth, the analysis relies on aggregated state–income cells and therefore cannot account for compositional changes within these groups over time, including shifts in age structure, racial and ethnic composition, educational attainment, or other characteristics associated with obesity prevalence. Future research using individual-level or sub-state data could provide more detailed insight into within-state variation and the interaction between income, geography, and obesity prevalence.

Finally, obesity prevalence is derived from self-reported height and weight. Although the first-difference specification reduces the influence of time-invariant reporting bias, it cannot eliminate bias arising from changes in reporting behavior over time or across population subgroups. If reporting accuracy varies differentially across income groups or periods, some residual measurement error may remain.

## Data Availability

Publicly available datasets were analyzed in this study. This data can be found here: https://www.cdc.gov/obesity/data-and-statistics/adult-obesity-prevalence-maps.html.
