# Supplementary material for: Variability of obesity prevalence in US states: are obesity growth or deterioration rates constant for different income quantiles?
Source: Front Epidemiol. 2026 Jul 8;6:1821610. doi: 10.3389/fepid.2026.1821610 (PMC13388406; doi:10.3389/fepid.2026.1821610)
Supplement: Supplementary file 1 [file Datasheet1.pdf]

Appendix A. Handling Self-Reported Measurement Error in First-Difference Specifications

Let the true (unobserved) obesity prevalence in state s, income quantile q, and year $t$be denoted by O^*_{sqt}. The observed obesity prevalence derived from BRFSS data, O_{sqt}, is based on self-reported height and weight and may therefore include systematic measurement error. We can write:

$$O_{sqt} = O^*_{sqt} + b_{sq} + \varepsilon_{sqt},$$

where b_{sq}captures time-invariant reporting bias specific to state $s$and income quantile q, and $\varepsilon_{sqt}$is a mean-zero idiosyncratic error term.

Our dependent variable is the annual percentage change in obesity prevalence:

$$\Delta O_{sqt} = O_{sqt} - O_{sq,t-1}.$$

Substituting the measurement equation yields:

Substituting the measurement equation yields:

$$\Delta O_{sqt} = \left(O^*_{sqt} - O^*_{sq,t-1}\right) + \left(b_{sq} - b_{sq}\right) + \left(\varepsilon_{sqt} - \varepsilon_{sq,t-1}\right).$$

The time-invariant bias term b_{sq} therefore differences out exactly, implying:

$$\Delta O_{sqt} = \Delta O^*_{sqt} + \Delta\varepsilon_{sqt}.$$

Under the assumption—supported by validation studies comparing BRFSS with objectively measured NHANES data—that systematic self-report bias in height and weight is relatively stable over moderate time horizons (Ezzati et al., 2006; Flegal et al., 2019), the first-difference specification yields consistent estimates of changes in true obesity prevalence. Consequently, while self-reported data may bias level estimates of obesity prevalence, such bias is unlikely to materially affect inference regarding income-specific trends and growth rates, which are the primary focus of this study.

Appendix B. Data Construction and Sample Composition

Appendix Table B1
Accounting of observations by jurisdiction – **extended sample (Income 0–5, N=2,826)**

This table provides an exact arithmetic reconciliation of the 2,826 observations used in the extended regression sample by grouping jurisdictions according to the number of observations they contribute.

Footnote	Number of jurisdictions	Observed observations per jurisdiction	Product (observations)
(1)	50	54	2700
(2)	1	48	48
(3)	1	42	42
(4)	1	36	36
Total	53		2826

Notes. The extended sample includes six income categories (0–5) and spans nine annual changes (2012–2020). A fully balanced jurisdiction contributes 54 observations (6 income categories × 9 annual changes). Jurisdictions with fewer observations reflect missing income-disaggregated obesity prevalence data required to compute Δ.

(1) Jurisdictions with 54 observations: Alabama, Alaska, Arizona, Arkansas, California, Colorado, Connecticut, Delaware, District of Columbia, Florida, Georgia, Hawaii, Idaho, Illinois, Indiana, Iowa, Kansas, Kentucky, Louisiana, Maine, Maryland, Massachusetts, Michigan, Minnesota, Mississippi, Missouri, Montana, Nebraska, Nevada, New Hampshire, New Mexico, New York, North Carolina, North Dakota, Ohio, Oklahoma, Oregon, Pennsylvania, Rhode Island, South Carolina, South Dakota, Tennessee, Texas, Utah, Vermont, Virginia, Washington, West Virginia, Wisconsin, Wyoming.

(2) Jurisdictions with 48 observations: New Jersey.

(3) Jurisdictions with 42 observations: Puerto Rico.

(4) Jurisdictions with 36 observations: Guam.

Appendix Table B2

Accounting of observations by jurisdiction – **baseline sample (Income 1–5, N=2,355)**

This table provides an exact arithmetic reconciliation of the 2,355 observations used in the regression analysis by grouping jurisdictions according to the number of observations they contribute to the estimation sample.

Footnote	Number of jurisdictions	Observed observations per jurisdiction	Product (observations)
(1)	50	45	2250
(2)	1	40	40
(3)	1	35	35
(4)	1	30	30
Total	53		2,355

Notes. This table provides an exact arithmetic reconciliation of the 2,355 observations used in the regression analysis by grouping jurisdictions according to the number of observations they contribute to the estimation sample. The underlying data span ten calendar years (2011–2020). Because the dependent variable Δis defined as the annual percent change in obesity prevalence and therefore requires a lagged prevalence measure, the first year of the sample (2011) is used solely to construct changes and is excluded from the estimation sample. **As a result, a fully balanced jurisdiction contributes 45 observations (5 income categories × 9 annual changes).** Jurisdictions with fewer observations reflect the absence of obesity prevalence data in one or more income categories or years required to compute Δ. There are no additional missing cells beyond this mechanical loss.

(1) Jurisdictions with 45 observations: Alabama, Alaska, Arizona, Arkansas, California, Colorado, Connecticut, Delaware, District of Columbia, Florida, Georgia, Hawaii, Idaho, Illinois, Indiana, Iowa, Kansas, Kentucky, Louisiana, Maine, Maryland, Massachusetts, Michigan, Minnesota, Mississippi, Missouri, Montana, Nebraska, Nevada, New Hampshire, New Mexico, New York, North Carolina, North Dakota, Ohio, Oklahoma, Oregon, Pennsylvania, Rhode Island, South Carolina, South Dakota, Tennessee, Texas, Utah, Vermont, Virginia, Washington, West Virginia, Wisconsin, Wyoming.

(2) Jurisdictions with 40 observations: New Jersey.

(3) Jurisdictions with 35 observations: Puerto Rico.

(4) Jurisdictions with 30 observations: Guam.

Appendix C: Robustness and Sensitivity Analyses

Appendix Table C1. Income Categories and Annual Change in Obesity Prevalence (Δ)

Dependent variable: Annual percentage change in obesity prevalence (Δ)

Sample: Income categories below $15,000 through $75,000+

Reference category: below $15,000

	(1)	(2)	(3)	(4)
	Robust SE	Robust SE	Clustered SE	Clustered SE
Variable	Full	Stepwise	Full	Stepwise
Constant	0.022233***	0.0255504***	0.022233***	0.024659***
	(0.006892)	(0.0021541)	(0.003051)	(0.001138)
Income $15,000–$24,999	-0.0022643	—	-0.002264	—
	(0.007735)		(0.003076)	
Income $25,000–$34,999	0.0041091	—	0.004109	—
	(0.008587)		(0.003279)	
Income $35,000–$49,999	0.0041925	—	0.004193	—
	(0.008183)		(0.003475)	
Income $50,000–$74,999	0.0026593	—	0.002659	—
	(0.008102)		(0.003278)	
Income $75,000 or greater	0.0070878	—	0.007088**	0.005348***
	(0.007766)		(0.003149)	(0.001866)
Year − 2012 (linear trend)	0.0001704	—	0.000170	—
	(0.000821)		(0.000614)	
Observations	2,826	2,826	2,826	2,826
States (clusters)	—	—	53	53
F-statistic	0.43	0.00	2.54	8.22
Prob > F	0.8598	0.0000	0.0314	0.0060

Notes. Columns (1) and (2) report heteroskedasticity-robust standard errors for the full and stepwise specifications, respectively. Columns (3) and (4) report the corresponding specifications with standard errors clustered at the state level. The full specification includes income category indicators and a linear time trend centered at 2012; the stepwise specification is selected using a removal criterion of $p < 0.05$. Statistical significance: *** $p < 0.01$, ** $p < 0.05$, * $p < 0.10$.

Appendix Figure C2: Predicted Annual Obesity Change by Income Category (Full Sample)

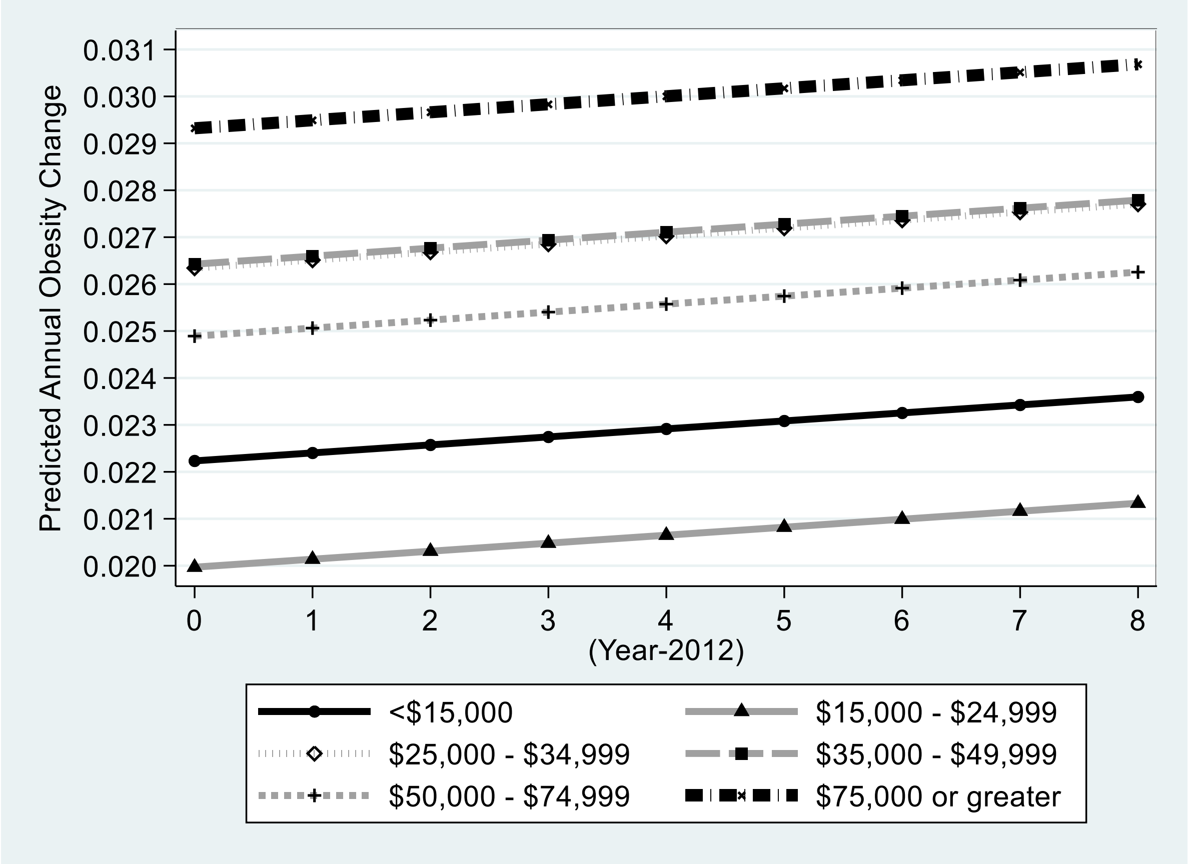

Notes. Predicted annual changes in obesity prevalence by household income category are computed from the full specification reported in column (3) of Appendix Table B1 (full sample), which includes income indicators and a linear time trend (year − 2012). The dependent variable is the year-to-year percentage change in obesity prevalence. The estimated coefficient on the time trend is not statistically significant. Predicted trajectories for the $25,000–$34,999 and $35,000–$49,999 income categories overlap closely over most of the sample period.

Appendix Figure C3: Predicted Annual Change in Obesity Prevalence by Income Category (Including <$15,000)

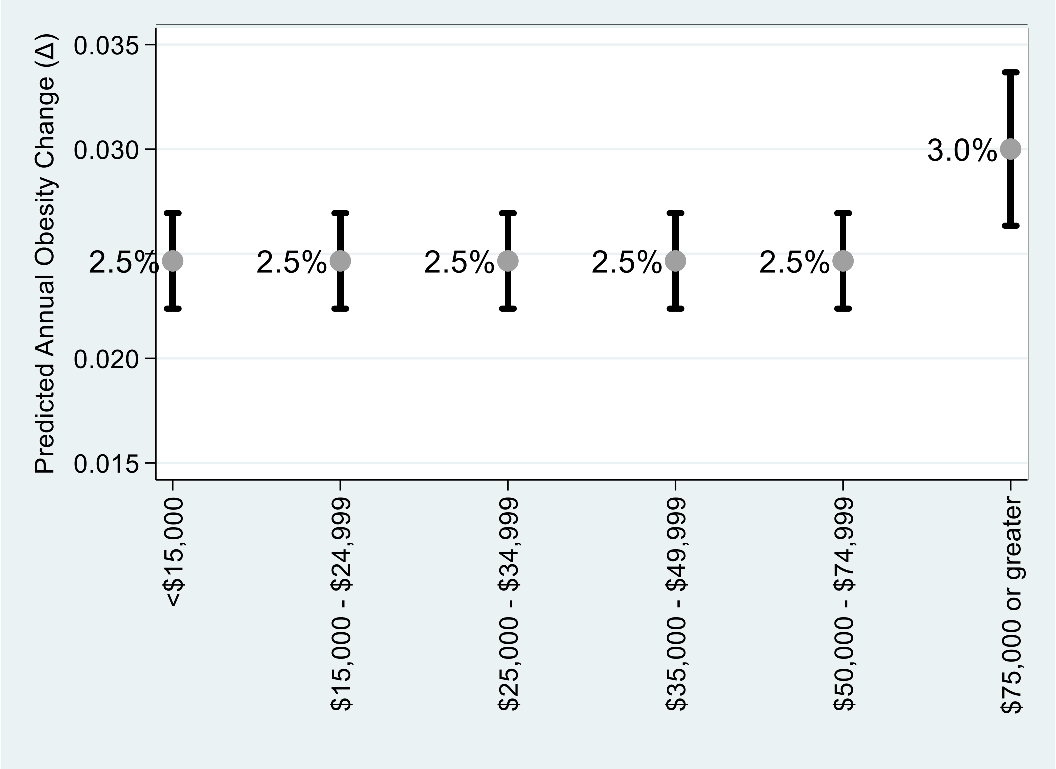

Notes. Predicted annual obesity changes by household income category are computed from the full specification reported in column (3) of Appendix Table B1, which includes income indicators and a linear time trend (year − 2012). The dependent variable is the year-to-year percentage change in obesity prevalence. Predicted values for households with incomes below $15,000 are nearly identical to those for households with incomes between $15,000 and $74,999, while the $75,000 or greater category exhibits a higher predicted annual change. The coefficient on the time trend is not statistically significant. This similarity provides motivation for excluding the <$15,000 category in the main-text analysis.

Appendix Figure C4: Differences in Annual Obesity Growth Relative to the <$15,000 Income Group

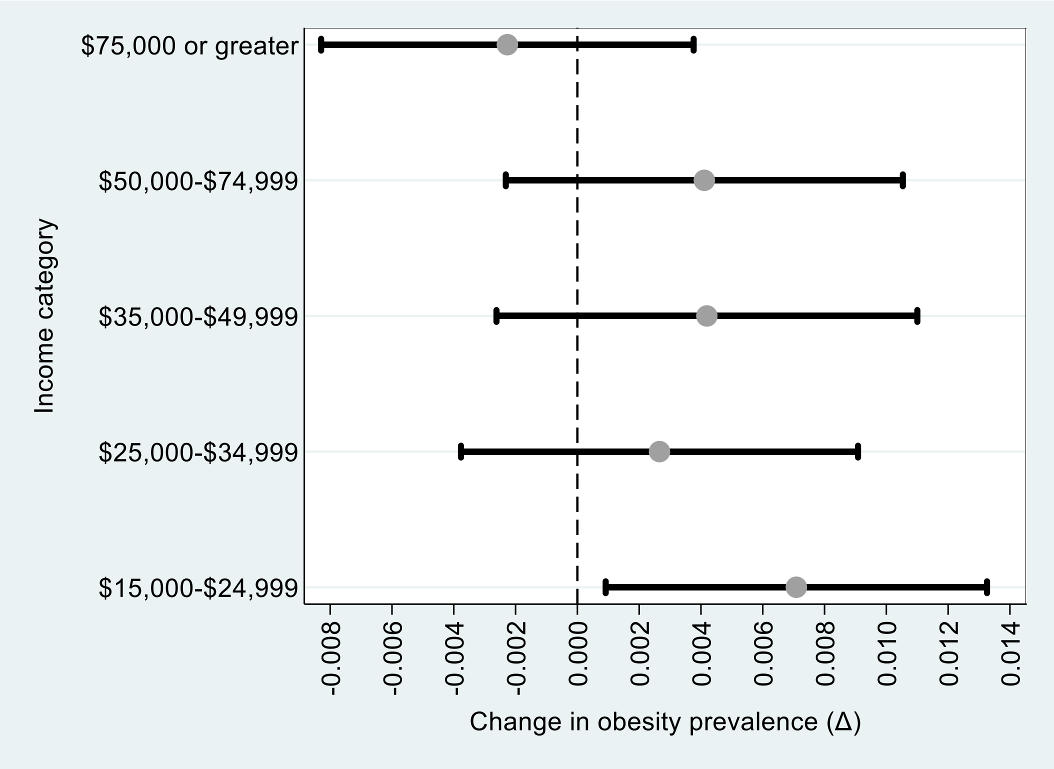

Notes. The figure shows estimated differences in the annual growth rate of obesity prevalence across household income categories, relative to the reference group with annual income below $15,000, based on the specification reported in column (3) of Appendix Table B1. Points denote coefficient estimates from a linear regression of the annual percentage change in obesity prevalence, and horizontal bars indicate 95% confidence intervals based on standard errors clustered at the state level. The dashed vertical line at zero denotes no difference relative to the reference category. The estimated annual growth rate of obesity prevalence for the reference group is 2.5%.

Appendix D. Robustness of Annual Change Estimates to Winsorization of Δ

Appendix Table D1. Robustness of Annual Change Estimates to Winsorization of Δ (1st/99th Percentiles)

Dependent variable: Annual percentage change in obesity prevalence (Δ), winsorized

Sample: Full sample (Income 0–5) and restricted sample excluding the lowest income group (Income 1–5)

Reference category: Income below $15,000 in column (1); Income $15,000–$24,999 in column (2).

	(1)	(2)
Variable	Clustered SE	Clustered SE
	Full sample (Income 0–5)	Restricted sample (Income 1–5)
Constant	0.11506***	0.11306***
	(0.00227)	(0.00256)
Income $15,000–$24,999	-0.00055	—
	(0.00258)	
Income $25,000–$34,999	0.00411	0.00465*
	(0.00292)	(0.00234)
Income $35,000–$49,999	0.00481*	0.00536**
	(0.00288)	(0.00244)
Income $50,000–$74,999	0.00442	0.00497**
	(0.00277)	(0.00200)
Income $75,000 or greater	0.00688***	0.00743***
	(0.00245)	(0.00192)
Year − 2012 (linear trend)	-0.01696***	-0.01655***
	(0.00055)	(0.00056)
Observations	3,162	2,635
States (clusters)	54	54

Notes. Δ denotes the annual percentage change in obesity prevalence. Δ is winsorized at the 1st and 99th percentiles. Standard errors clustered at the state level are reported in parentheses. Statistical significance: *** $p < 0.01$, ** $p < 0.05$, * $p < 0.10$.

Appendix Table E1. Linear Regression Estimates

Dependent variable: Percentage

Sample: Full sample and restricted sample excluding the lowest income group

Reference category: Income below $25,000 in column (1); Income $15,000–$24,999 in column (2).

Variable	(1)<br>Clustered SE<br>Full sample	(2)<br>Clustered SE<br>Restricted Sample
Constant	0.4500***	0.4317***
	(0.0885)	(0.0934)
Income $15,000–$24,999	0.0454	—
	(0.0970)	
Income $25,000–$34,999	0.1493	0.1038
	(0.0966)	(0.0888)
Income $35,000–$49,999	0.1620	0.1166
	(0.0983)	(0.0763)
Income $50,000–$74,999	0.1183	0.0728
	(0.0939)	(0.0644)
Income $75,000 or greater	0.1890**	0.1435**
	(0.0830)	(0.0612)
Year – 2012	0.0095	0.0253
	(0.0177)	(0.0175)
Observations	2,826	2,355
States (clusters)	53	53

Notes: Robust standard errors clustered by state in parentheses. ** $p<0.05$, *** $p<0.01$.
